# Herbal medicines in functional dyspepsia—Untapped opportunities not without risks

**DOI:** 10.1111/nmo.14044

**Published:** 2020-11-30

**Authors:** Kok‐Ann Gwee, Gerald Holtmann, Jan Tack, Hidekazu Suzuki, Jinsong Liu, Yinglian Xiao, Min‐Hu Chen, Xiaohua Hou, Deng‐Chyang Wu, Clarissa Toh, Fang Lu, Xu‐Dong Tang

**Affiliations:** ^1^ Department of Medicine Yong Loo Lin School of Medicine National University of Singapore and Gleneagles Hospital Singapore City Singapore; ^2^ Faculty of Medicine & Faculty of Health & Behavioural Sciences University of Queensland and Department of Gastroenterology & Hepatology Princess Alexandra Hospital Woolloongabba Queensland Australia; ^3^ Department of Gastroenterology University Hospitals Leuven Leuven Belgium; ^4^ Division of Gastroenterology and Hepatology Department of Internal Medicine Tokai University School of Medicine Tokyo Japan; ^5^ Gastroenterology Department Wuhan Union Hospital Huazhong Science & Technology University Wuhan China; ^6^ Division of Gastroenterology and Hepatology The First Affiliated Hospital Sun Yat‐sen University Guangzhou China; ^7^ Division of Gastroenterology Wuhan Union Hospital Huazhong Science & Technology University Wuhan China; ^8^ Division of Gastroenterology Department of Internal Medicine, and Department of Medicine College of Medicine Kaohsiung Medical University Kaohsiung Taiwan; ^9^ Independent Researcher Stomach, Liver & Bowel Centre Gleneagles Hospital Singapore City Singapore; ^10^ Xiyuan Hospital China Academy of Chinese Medical Sciences Beijing China

**Keywords:** functional dyspepsia, gastrointestinal physiology, herbal medicine, pharmacology, toxicity, treatment algorithms

## Abstract

**Background:**

Contemporary treatments for functional dyspepsia have limitations. Herbal medicine has been suggested as adjunctive treatment. With growing scientific recognition and public interests, an in‐depth review of this is timely.

**Aims/Purpose:**

To evaluate the therapeutic potential and problems that may be associated with the adoption of herbal medicines in functional dyspepsia.

**Methods:**

We reviewed the treatment landscape of functional dyspepsia and assessed the scientific community's interest in herbal medicine. Preclinical pharmacological and clinical trial data were reviewed for several herbal medicines available in the market. Challenges associated with adoption of herbal medicine in mainstream medicine were critically evaluated.

**Results:**

We found that herbal medicines frequently comprise a combination of herbs with multiple reported pharmacological effects on gastrointestinal motility and secretory functions, as well as cytoprotective and psychotropic properties. We identified a number of commercially available herbal products that have undergone rigorous clinical trials, involving large numbers of well‐defined subjects, reporting both efficacy and safety for functional dyspepsia. Persisting concerns include lack of rigorous assessments for majority of products, toxicity, consistency of ingredients, dose standardizations, and quality control. We provide a quality framework for its evaluation.

**Conclusions:**

We commend herbal medicine as a viable future option in managing functional dyspepsia. An attractive appeal of herbal medicine is the prospect to simultaneously target multiple pathophysiological mechanisms. Wider adoption and acceptance of herbal medicines in treatment algorithms of functional dyspepsia will require the application of the scientific rigor expected of chemical therapies, to all stages of their development and evaluation.

**Key Points**

- Chemically defined therapeutic targets appear inconsistent with the multifactorial nature of functional dyspepsia.
- Mechanistic studies indicate that herbs individually and in combination have multiple concurrent pharmacological activities relevant to dyspepsia. Meta‐analyses provide signals of efficacy and safety for dyspepsia with several commercially available herbal compounds found to have at least RCTs published in mainstream journals.
- Herbal medicines expand the scope for the treatment of functional dyspepsia, an underserved therapeutic area.

## 1 TREATMENT LANDSCAPE IN FD

Therapeutic options for FD are currently limited and include prokinetics, antisecretory agents, eradication of *Helicobacter pylori*, and centrally acting neuromodulators.^1^ With gains over placebo not exceeding 10–20%, the efficacy is limited (see Table 1).^1^, ^2^ Conventional chemically defined treatment approaches ideally target specific more or less well‐characterized pathophysiologic disease mechanisms while many first‐line treatments are recommended based upon specific symptom clusters (e.g., acid blockers in patients with epigastric pain syndrome (EPS) or prokinetic agents for the postprandial distress syndrome (PDS).^2^

**TABLE 1:** Efficacy and limitations for different treatment classes in FD (adapted from Masuy 2019)^1^

Treatment class	Efficacy	Limitations
PPI	30–70%	Exact mechanism of action unclear, efficacy controversial with conflicting results
Dopamine‐2 antagonist	59–81%	Controversial effect on gastric emptying versus symptom relief. Extrapyramidal symptoms. Domperidone: Cardiac safety
5HT4 agonist	32–91%	Beneficial effects for certain subgroups. Cardiac safety concerns. Controversial effect on gastric emptying vs. symptom relief
Muscarinic antagonist	31–80%	Only 2 studies comparing efficacy for PDS & EPS subgroups, with conflicting results
*Helicobacter pylori* eradication	24–82%	Effects of eradication therapy on gastric function and the mechanism of actions are unclear. High number needed to treat (NTT). The effect could also be related to non‐*H. pylori*‐related antimicrobial or anti‐inflammatory effects
Neuromodulator	27–71%	Limited trials in FD, Efficacy and exact MOA in FD unclear. TCA: Side effects concern
Psychotherapy	38–63%	? cost effectiveness, lack of trained personnel, time consuming, lacking high‐quality studies

"? cost effectiveness" = uncertain cost effectiveness due to variations in availability and insurance coverage.

Possible explanations for the limited effects of available chemically defined therapies include the heterogeneity of putative pathophysiologies.^3^ In addition, the links between disturbed functions and symptoms (e.g., gastric emptying and postprandial fullness) are weak, and consequentially, there is a lack of robust predictors of response.^1^, ^4^ Substantial numbers of patients also fulfill both EPS and PDS criteria by the Rome III criteria, rendering a subgroup‐based initial therapy recommendation ineffective.^5^ Thus, multiple pathophysiological disturbances may co‐exist in patients who manifest with more than one symptom cluster. Early data suggest that the Rome IV criteria may produce a sharper discrimination among the different subgroups.^6^, ^7^ However, this has yet to be substantiated in clinic populations.^8^

Furthermore, in the outpatient clinic there is substantial overlap of FD with other functional gastrointestinal disorders (FGID) syndrome. In a pan‐Asian survey of primary and secondary care GI clinics, an estimated 83% of FD (by Rome III) had an overlapping condition.^9^ This was reinforced by a study from Australia where in a tertiary center, the majority of FGID patients had overlapping FD and irritable bowel syndrome (IBS).^10^ Importantly, FD patients who have overlapping symptoms are more difficult to treat; they report greater symptom severity, treatment dissatisfaction, more physician visits, specialist referrals, and surgery.^11^, ^12^, ^13^, ^14^, ^15^

## 2 HERBAL MEDICINE AS A THERAPEUTIC OPTION IN FGID

The therapeutic potential of herbal medicine in general is gaining recognition. The World Health Organization's (WHO) recent 11th revision of International Classification of Diseases (ICD‐11) included details about Traditional Chinese Medicine (TCM) for the first time as part of their WHO Traditional Medicine Strategy (2014–2023).^16^

**FIGURE 1:**
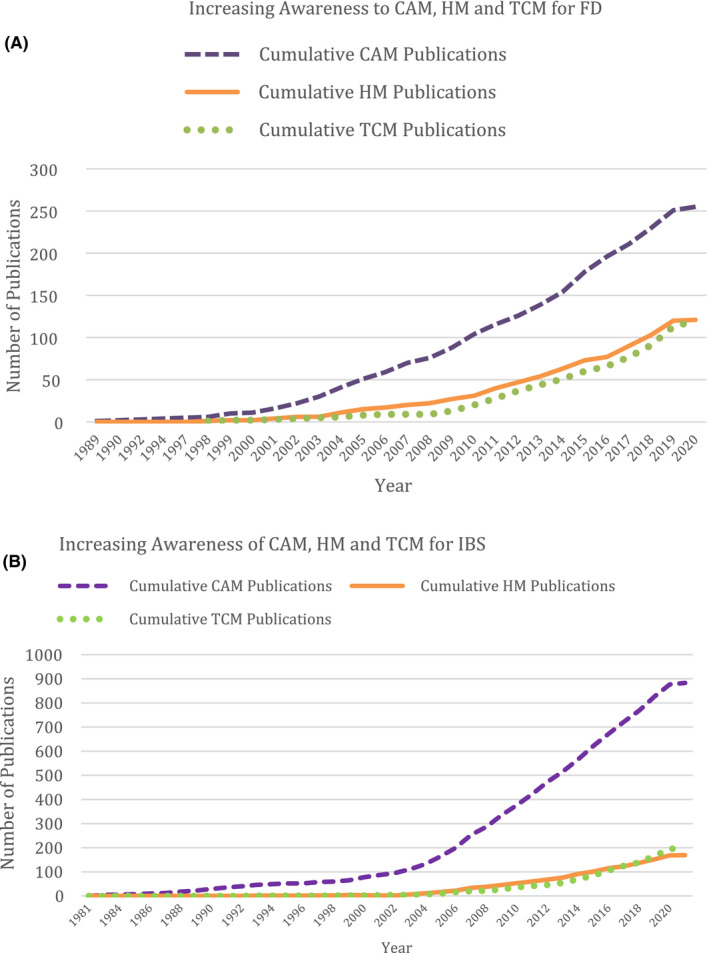
A, Publication charts showing increasing number of publications for **functional dyspepsia**. B, Publication charts showing increasing number of publications for **irritable bowel syndrome**. Source: PubMed. (As of March 8th 2020). Abbreviations: CAM, Complementary and Alternative Medicine; HM, Herbal Medicine; TCM, Traditional Chinese Medicine

Global interest in the role of herbal medicines for FGIDs has grown in recent years attested by the increasing publications pertaining to herbal medicines. (Figure 1A on FD and 1B on IBS).

While herbal medicine may be regarded as one component of complementary alternative medicine (CAM), some herbal formulations have become part of mainstream medicine. For example, the use of peppermint oil as relief of abdominal colic and distension, particularly in IBS, is recommended in the British National Formulary (BNF).^17^ CAM treatments can range from acupuncture and homeopathy, to meditation and colonic irrigation.^18^ According to a 2012 survey by US National Center for Complementary and Integrative Health, natural products,^a^ deep breathing, yoga/*Tai Chi*/*Qi Gong* and chiropractic/osteopath are the top complementary health approaches among adults.^19^

In Italy, a study of GI outpatient clinics found that 36.7% of FGID patients had used herbal drugs, whereas only 8% had received antidepressants.^20^ Furthermore, two‐thirds would use a combination of treatment modalities. A study of patients with functional bowel disorders in the USA found a CAM usage of 35%.^21^ Dissatisfaction with their physicians or with conventional medicines did not appear to be a factor driving use of CAM, while female gender, college education, and anxiety were positive predictors. However, a study from Australia found that seeking care from an alternative healthcare provider was not related to psychological morbidity.^22^

While in the Western world the utilization is patchy,^23^ in Asia, there is a long tradition of use and high acceptance, of herbal medicine. For example, *Liu Jun Zi Tang* (LJZT) (known in Japan as Rikkunshito), a traditional herbal medicine which has been used to treat dyspepsia, was described as early as the 16^th^ Century.^24^ In Taiwan, Chinese herbal medicines (CHM) are covered under their national health insurance, with constipation and functional disorders of stomach two of the conditions receiving the highest prescription of CHM.^25^, ^26^ In a Taipei general hospital, of 50 patients attending a GI outpatient clinic, traditional herbal medicines had been used by one‐third, with 85% using in combination with conventional Western medications.^27^

## 3 POTENTIAL OF HERBAL MEDICINES IN FD—PRECLINICAL AND CLINICAL STUDIES

**FIGURE 2:**
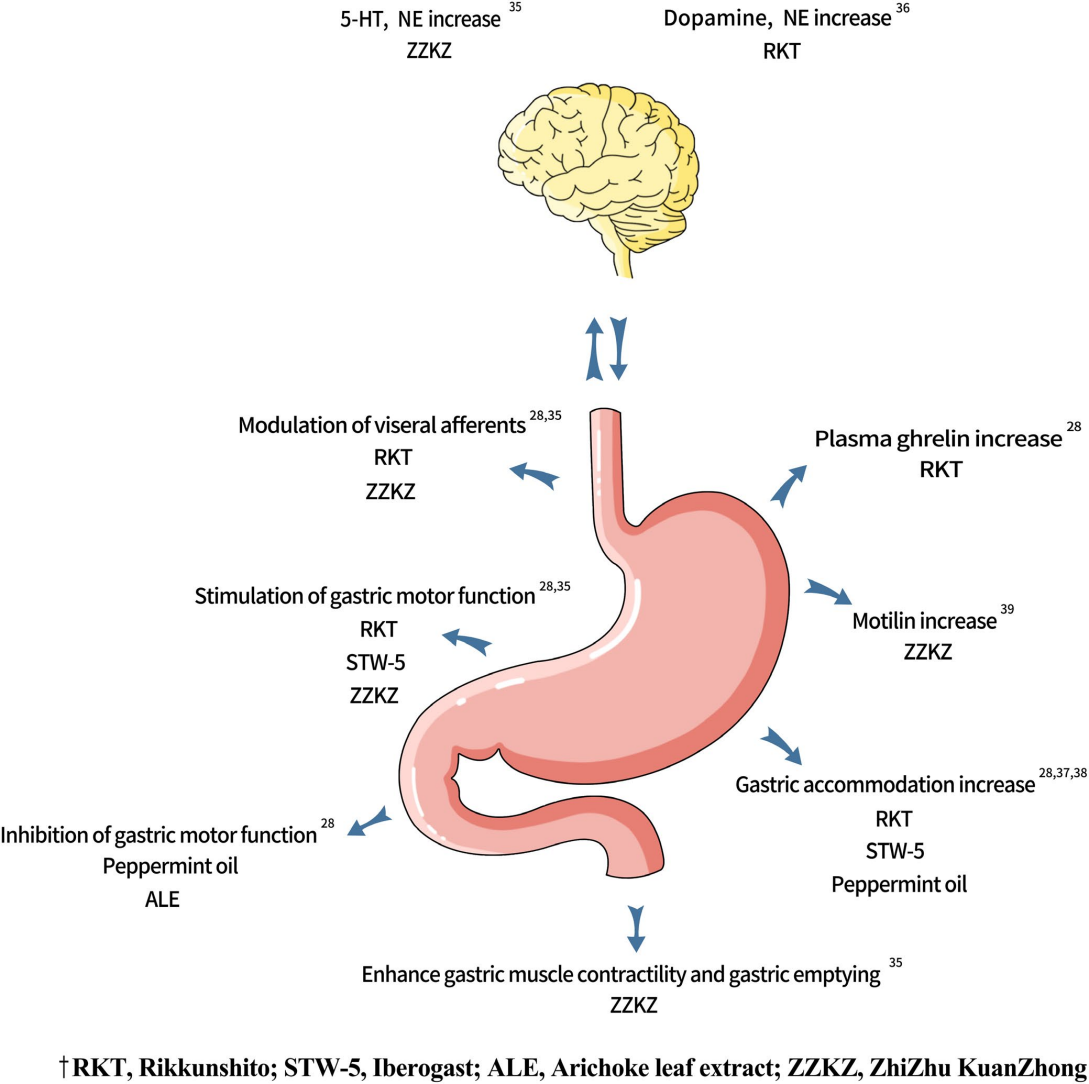
Herbal compounds for FD: pharmacological effects on gastric functions.^28^, ^35^, ^36^, ^37^, ^38^, ^39^ Abbreviations: ALE, Artichoke leaf extract; RKT, Rikkunshito; STW‐5, Iberogast; ZZKZ, ZhiZhu KuanZhong

A recent review proposed to position herbal medicines as adjunctive therapy that could be introduced at all levels (primary to tertiary) in the management algorithm of FD.^1^ An attractive appeal of herbal medicines is the prospect to target simultaneously multiple putative pathophysiological mechanisms. Herbal medicines frequently comprise a combination of herbs with multiple reported effects on gastrointestinal motility, secretory functions, and cytoprotective and even psychotropic properties.^23^, ^28^, ^29^, ^30^, ^31^ Cremonini (2014) had described the multiple putative therapeutic properties relevant to gastric functions for peppermint oil, artichoke leaf extract, STW‐5, and Rikkunshito.^28^ Recently, laboratory and clinical studies have also described multiple relevant pharmacological effects for herbal medicines (Figure 2).

The pharmacological effects of herbal compounds, or their component herbs, on putative pathophysiology of FD were largely studied in bench and preclinical settings. Their modes of actions may not have been examined in the relevant patient subjects. Furthermore, doses employed in mechanistic studies may have differed from those used in the clinical trials. (The differences between doses used in preclinical and clinical studies are elaborated in section 3.1 on STW‐5).

Among the herbal formulations studied for FD, we found specific systematic reviews for Rikkunshito and peppermint oil–caraway oil. For both compounds, the data were found to be inconclusive for efficacy due to the limited number of studies considered to be at low risk of bias.^24^, ^32^, ^33^ While we recognize the limitations of systematic reviews where different herbal medicine formulations are collectively analyzed, this approach serves to at least provide a signal of the therapeutic and safety potential for herbal medicine as a treatment class. In this respect, a systematic review of clinical trials in FGIDs, efficacy was found to be in favor of herbal medicines over placebo (RR = 1.53, 95% CI 1.34–1.75). With regard to safety, while herbal medicines were found to have a higher risk of adverse events over placebo (RR = 1.29, 95% CI 1.12–1.49), the risk was similar to conventional drugs (RR = 0.91, 95% CI 0.71–1.17). Subgroup analysis found that specifically for FD, herbal medicines were better than placebo in alleviating symptoms (RR = 1.51, 95% CI 1.33–1.71).^34^

The preclinical and clinical data for herbal medicine formulations with relevant effects for FD where there are at least randomized clinical trial data published in mainstream journals, will be briefly reviewed in terms of (a) findings from preclinical studies, (b) systematic reviews if available, (c) findings from key clinical studies, and (d) study limitations.

### 3.1 STW‐5 (IBEROGAST^®^)

The combination herbal formulation known as STW‐5 (Iberogast^®^, Steigerwald Arzneimittelwerk GmbH, Darmstadt, Germany) is a fixed combination of 9 different herbs, *Iberis amara totalis recens, Angelicae radix, Cardui mariae fructus, Chelidonii herba, Liquiritiae radix, Matricariae flos, Melissae folium, Carvi fructus,* and *Menthae piperitae folium*.^40^ STW‐5 was demonstrated to produce relaxation of muscles from the gastric fundus (which could improve fundus accommodation), while at the same time to increase contractility of muscles from the gastric antrum. *Iberis amara*, or Bitter candy tuft, is purported to have multiple effects, such as stimulatory effect on smooth muscles of the stomach and small intestine, reduction of leukotriene concentration and acid secretion, and dose‐dependent antiulcerogenic effect,^21^, ^23^ while *Matricariae flos*, better known as chamomile flower, is reported to have antidepressive and anxiolytic effects.^23^ In a preclinical study of 12 healthy subjects, STW‐5 administered at 1.1 ml single dose daily was associated with increased proximal gastric volumes and antral motility but did not appear to affect pyloric or duodenal motility, gastric emptying of solids and liquids or intragastric distribution of test meals.^28^ In clinical studies of FD patients, the dose used was much higher at 3 × 20 drops/day (Total 3 ml daily), similar to the recommended dose.

There are three published double‐blind, placebo‐controlled studies showing superiority of STW‐5 in improving symptoms in FD. In a relatively small study, 60 patients were treated for 4 weeks with STW‐5 after a 6‐day run‐in without medication. Patients treated with STW‐5 experienced a significantly better improvement of the symptoms score.^41^ In a randomized crossover study, 120 FD patients are given three 4‐week treatment blocks. During the first 4 weeks, the Gastrointestinal Symptom Score (GIS) significantly decreased in subjects on active treatment compared to the placebo (*p* < 0.001). After 8 weeks, 43.3% on active treatment and 3.3% on placebo reported complete relief of symptoms. (*p* < 0.001 vs. placebo).^21^ Another study aimed to explore the effect of a concomitant *H. pylori* infection on the response to STW‐5. In this study, a 7‐day run‐in phase was required prior to treatment. A total of 315 patients were treated with 3 × 20 drops/day, of either STW 5 or placebo. Symptom assessment was done at baseline and at 2, 4, and 8 weeks of treatment. The principal outcome criterion was the change in a validated GIS. The STW‐5 group improved significantly more as compared to controls (*p* < 0.05), and *H. pylori* did not influence the results.^42^ As all three studies had not pre‐specified the FD subtype, and all had applied a composite GIS, it is not possible to ascertain whether there is any symptom‐ or subtype‐specific response.

### 3.2 Peppermint oil–caraway oil (POCO)

Peppermint oil is extracted from the leaves of *Mentha piperita* *L*., and the peppermint oil and caraway oil combination is available commercially as a proprietary formulation known as Menthacarin. Peppermint oil has calcium antagonistic properties that could induce relaxation of smooth muscles^43^—promoting increased gastric accommodation and having antispasmodic action in the intestine. Similarly, smooth muscle relaxant effect has also been reported for caraway oil, and its combination with peppermint oil (POCO) has reported effects on gastric and gallbladder emptying, and modulating visceral hypersensitivity.^32^, ^43^, ^44^, ^45^, ^46^, ^47^ Notwithstanding, a meta‐analysis of five POCO RCT studies shows that POCO can significantly improve global symptoms of FD, with safety similar to placebo.^32^ Three placebo‐controlled RCTs reported efficacy POCO for reducing FD symptoms with effects for both epigastric pain syndrome (EPS) and postprandial distress syndrome (PDS) subsets.^48^, ^49^, ^50^ However, mechanistic studies to verify the effects of this combination in patients with dyspepsia are lacking.^1^

### 3.3 Rikkunshito

Rikkunshito (RKT), also known by its Chinese name of *Liu Jun Zi Tang* (LJZT), consists of eight major constituents herbs—*Atractylodis lanceae rhizoma, Ginseng radix, Pinelliae tuber, Poria, Zizyphi fructus, Citri unshiu pericarpium, Glycyrrhizae radix, and Zingiberis rhizoma*.^51^, ^52^

RKT contains several active compounds that work on gastric functions (Figure 2). Atractylodin, extracted from *Atractylodis lanceae rhizoma*, was demonstrated to have ghrelin signal enhancement effect.^28^, ^51^ Ghrelin is a peripheral hormone which is involved in appetite stimulation and modulation of several GI functions.^51^, ^52^ Hesperidin, a major active compound in RKT extracted from *Citri unshiu pericarpium*, exerts its effect on gastric emptying through suppression of serotonin receptors.^53^

In a recent large systematic review and meta‐analysis for upper gastrointestinal symptoms (24 studies with a combined total of 2175 participants), RKT significantly relieved upper GI symptoms on a 5‐point scale but was insignificant when compared with other treatments.^33^ Another meta‐analysis focusing on FD showed that RKT compared with prokinetic drugs increased dyspeptic symptom improvement.^24^ No adverse reaction for RKT was recorded in both studies. In recent years, there are six RKT RCTs for FD with close to 1000 FD patients involved.^54^, ^55^, ^56^, ^57^, ^58^, ^59^ One Japanese study reported that RKT significantly improved epigastric pain (*p* = 0.04), especially in *H. pylori*‐infected patients (RKT 40.0% vs. placebo: 20.5%, *p* = 0.07), and seemed less effective among *H. pylori*‐uninfected participants^55^ (RKT: 29.3% vs. placebo: 25.6%, *p* = 0.72), while another Japanese RCT showed non‐consumption of alcohol was associated with the efficacy of RKT especially among *H. pylori*‐infected participants.^56^ The two Chinese studies demonstrated that RKT patients achieved significant improvements in gastric emptying and symptom scores (TSS, SDS, PDSS, and CGI scale^b^) compared to placebo.^54^, ^57^ In the pilot study in Europe, it was concluded that treatment with RKT improved upper GI symptoms in FD patients but similarly high placebo effects were observed.^55^

As summarized in Table 2, RKT has been reported to have various centrally acting effects for FD patients. Besides being an initial treatment option for Anorexia Nervosa via facilitation of ghrelin secretion,^34^, ^60^ two RCTs reported RKT subjects achieved improvement in Hospital Anxiety and Depression Scale (HADS)^56^, ^57^ versus placebo after 4 and 8 weeks of RKT treatment, respectively. RKT has also been shown to improve psychological stress by various pathways, such as attenuating the activities of corticotropin‐releasing hormone (CRH)‐producing neurons, leading to lowered anxiety‐like behavior in tumor‐bearing rats.^60^ Stress markers such as adrenocorticotropic hormone, cortisol, and neuropeptide Y were also lowered by RKT in blood samples of healthy subjects.^61^ RKT is also reported to ameliorate cancer anorexia–cachexia syndrome, mediated by synergistically promoting endogenous ghrelin activity by several components of RKT^62^ and elevation of glucarate levels in tumor‐bearing rats.^63^

**TABLE 2:** Herbal compounds for FD: key ingredients, preclinical pharmacological effects, and clinical trial data

Preparation	Key ingredients	Key clinical trial data outcome	Purported effects on GI functions	Purported effects on central functions
STW‐5	Iberis amara totalis recens, Angelicae radix, Cardui mariae fructus, Chelidonii herba, Liquiritiae radix, Matricariae flos, Melissae folium, Carvi fructus and Menthae piperitae folium	Significant improvement of gastrointestinal symptom score (GIS), global effectiveness, and tolerability	Increase gastric accommodation, modulation of gastric sensorimotor function, nociception, bile and gastric acid clearance	
RKT	*Atractylodis lanceae rhizoma, Ginseng radix, Pinelliae tuber, Poria, Zizyphi fructus, Citri unshiu pericarpium, Glycyrrhizae radix, and Zingiberis rhizoma*	Improve epigastric pain, especially for *Helicobacter pylori*‐infected patients	Modulation of visceral afferents (sensory), increase gastric accommodation, increase plasma ghrelin, stimulation of gastric motor function	Improvement in Hospital Anxiety and Depression Scale (HADS), lowered anxiety‐like behavior, reduced stress markers, and ameliorate cancer anorexia–cachexia syndrome
ALE	Bitter compounds (cynaropicrin)	Overall symptom improvement and greater improvement in QoL	Increase bile flow exerting hepatoprotective, antioxidant and antispasmodic effects	
POCO	Leaf Extract of *Mentha piperita* L.	Reducing FD symptoms with effects for EPS and PDS subsets	Increase gastric accommodation, inhibition of gastric motor function	
ZZKZ	*Atractylodes macrocephala (Bai Zhu), Citrus aurantium (Zhi Shi), Bupleurum (Chai Hu), and Crataegus pinnatifida or Hawthorn (Shan Zha)*	Relieving postprandial fullness and early satiety	Accelerate gastric emptying and intestinal propulsion rate and mobility, stimulation of gastric motor function	Reduce depression and anxiety scores

Abbreviations: ALE, Artichoke leaf extract; POCO, peppermint oil and caraway oil; QoL, quality of life; RKT, Rikkunshito; STW‐5, Iberogast; ZZKZ, ZhiZhu KuanZhong.

### 3.4 Artichoke leaf extract (ALE)

Artichoke (*Cynara scolymus*) leaf extract (ALE) has traditionally been used to treat FD symptoms. ALE has been reported to possess inhibitory activity to the contractile response elicited by acetylcholine in animal ileum.^28^ The bitter compound, cynaropicrin, is believed to be responsible for the effects such as increase bile flow, leading to hepatoprotective, lipid‐lowering, antioxidant, and antispasmodic actions.^28^ In a large RCT with 244 FD patients, ALE demonstrated superior symptom alleviation (*p* < 0.001) and improved disease‐specific quality of life (Nepean Dyspepsia Index) compared to placebo. Patients reported symptom improvement on ALE as early as the first week of therapy.^64^

### 3.5 Zhizhu Kuanzhong (ZZKZ)

Like STW‐5, Menthacarin, and RKT, Zhizhu Kuanzhong (ZZKZ; Lonch Group Shanxi Shuang Ren Pharmaceuticals Co. Ltd, Shangxi Province, China) is a commercially available proprietary fixed combination formulation which has been on the market since 2002 in China. It consists of four herbs*—Atractylodes macrocephala* (*Bai Zhu*), *Citrus aurantium* (*Zhi Shi*), *Bupleurum* (*Chai Hu*), and *Crataegus pinnatifida* or Hawthorn (*Shan Zha*).^65^ In animal pharmacological studies, the main ingredient *Shan Zha and Bai Zhu* have been studied and were reported to promote gastric emptying and intestinal propulsion rate compared to control treatment.^66^, ^67^ *Zhi Shi* is commonly used on its own as a TCM for FD treatment.^68^ In *in vitro* experiments, *Zhi Shi* exhibited inhibitory action on the spontaneous contraction of pyloric circular smooth muscle strip.^68^ *Chai Hu* has been proven to enhance gastric fluid emptying and small intestinal transit speed, with an antianxiety and antidepressant effect.^65^ As a proprietary formula, ZZKZ demonstrated increased gastric emptying and intestinal propulsion and mobility in rats.^35^, ^65^, ^66^ A meta‐analysis on the effect of ZZKZ for FD found that ZZKZ alone or combined with routine western medicine showed a better efficacy compared with the control group of western medicine only (OR =3.32, 95% CI 2.66–4.15).^65^

In what is possibly the largest study (392 FD patients) to date of any multicenter double‐blind RCT herbal medicines for FD, ZZKZ at 3 × 2 capsules daily was shown to be superior to placebo for patients with Rome III PDS criteria.^65^ In particular, ZZKZ improved postprandial fullness and satiation. This appears to be a safe drug as no major adverse effect had been reported in the various RCTs and post‐marketing surveillance records. ^65^, ^66^

In a small Phase II, RCT with 403 FD subjects divided into three groups: double‐blinded ZZKZ, double‐blinded cisapride (an established prokinetic that has been withdrawn due to cardiotoxicity), and open‐treatment ZZKZ group, ZZKZ at 3 × 3 capsules daily was shown to be non‐inferior to cisapride in FD symptom control and gastric emptying.^67^ In an observation trial, ZZKZ at the same dosing of 3 × 3 capsules daily had similar effects as a combination of domperidone and St John's wort in reducing depression and anxiety symptom scores of FD patients.^68^

A compilation of reported pharmacological properties is summarized in Table 2.

### 3.6 Prospect for treating FD‐IBS overlap

STW‐5 and Menthacarin have experimental, preclinical, and clinical studies that suggest pharmacological actions in the intestine in addition to their effects in the stomach. STW‐5 has reported antispasmodic effects on intestinal smooth muscles, abrogation of enteric afferent nerve sensitivity, and prosecretory effects, all of which could be useful in treating the IBS.^69^, ^70^, ^71^, ^72^ There is one double‐blind placebo‐controlled trial in IBS, reporting superiority for STW‐5.^73^ A recent re‐evaluation of data from three early clinical studies with Menthacarin found that some of the FD subjects in these trials had overlapping IBS symptoms of diarrhea and flatulence, which had also improved during treatment with Menthacarin.^74^ In addition, peppermint oil by itself has reported efficacy in IBS.^75^, ^76^

## 4 PROBLEMS AND CHALLENGES WITH HERBAL MEDICINE

The following are specific issues pertaining to the adoption of herbal medicine that we have identified: lack of rigorous assessments, toxicity and safety concerns, drug formats, consistency of ingredients, and dosage standardization and quality control, and translation from traditional to contemporary health systems.

### 4.1 Lack of rigorous assessments

The lack of rigorous assessments and clear evidence for TCM have been some of the reasons cited by critics against its inclusion into ICD‐11.^77^ The European Academies Science Advisory Council (EASAC) and Federation of European Academies of Medicine (FEAM) have stated categorically that TCM‐originated compounds and other CAM should be reviewed critically before use, and should be subjected to the same rigorous assessment, that is, high‐quality RCTs.^78^ An evaluation of the available clinical trials of herbal medicines in FGID found significant risk of bias; out of fifty trials reviewed, only nine had low risk of bias. Four trials had high risk, and thirty‐six trials had unclear risks due to poor design. The authors concluded that the high risk of bias from the trials could be due to limitations in RCT design such as paucity of detailed methodology, non‐standardized evaluation of efficacy, and the suboptimal quality of the study design. The most frequent methodological deficiencies identified in these trials were in the methods of allocation concealment, the blinding of outcome assessment, and incomplete outcome data of AEs.^34^

### 4.2 Toxicity and safety concerns of herbal medicine

Probably the strongest reservation that health authorities and mainstream Western medicine‐trained physicians have with regard to herbal medicines is the risk of toxicity. A classic example is digitalis, derived from foxglove plants to produce digoxin for treatment of heart failure and arrhythmia.

In a study from Taiwan involving a million patients, it was found that 14% had received conventional drugs and CHM on the same day; 94% of these patients had received their prescriptions from different locations, with a high likelihood that prescribers were unaware of this co‐prescription, thus increasing the risk of drug interactions.^79^ Worryingly, in another study from Taiwan, two‐thirds had not informed their doctors of this dual practice.^27^

In general herbal medicines are one of the commonest treatment class implicated in liver failure.^80^, ^81^ Greater celandine, found in the herbal combination STW‐5, has been reported to be linked with acute liver injury in a published case report from Europe, suggesting the potential hepatotoxicity of Greater Celandine.^82^ However, controlled clinical trials of STW‐5 in FD have not reported any severe adverse events (SAE) in line with post‐marketing surveillance studies of STW‐5 between 1990 and 2013 where 80 million patients have been treated.^40^, ^83^

In a recent study of causes of acute‐on‐chronic liver failure (ACLF) in Asia, drugs were implicated as a cause in 10.5%, but within this category, CAM accounted for 71.7%.^84^ Based on studies from China, CHM were the commonest identifiable cause for liver failure.^85^, ^86^ According to the latest China's National Adverse Drug Reaction Monitoring Report 2019, over 1.5 million drugs comprising of western medicine, biological products and CHM were reported for adverse drug reactions from 1999 to 2019. Of these, CHM comprised 12.7% of the reported drugs, compared to 84.9% of chemically derived drugs. Furthermore, out of the 200,000 SAE reported in 2019 alone, CHM made up only 7.1%, suggesting the overall safety of CHM.^87^

Similar to virtually all chemically defined treatments, plant extracts or herbal medicines also can cause adverse reactions. Adverse reactions can be due to toxic effects of active ingredients, toxic effects of contaminants that have not been identified or eliminated during the production process, or idiosyncratic drug reactions. With appropriate measures, the risks due to toxic effects of active ingredients and toxic effects of contaminations can be mitigated. However, the risk of very rare idiosyncratic reactions cannot be completely ruled out. Thus, appropriate pharmacovigilance must be considered mandatory for herbal medicines. Based on currently available published data in FD, it appears that the adverse event profiles of herbal medicines are not greater than conventional medicines. (see Table 3) While reported numbers appear low, we express reservations in view of the reports on contribution of herbal medicine to SAEs like liver failure. The commonest side effects of herbal medicine were GI related such as abdominal pain, diarrhea, and nausea, not unlike the side effects of some contemporary FD medications such as domperidone.

**TABLE 3:** Incidence of adverse events (AE) and adverse reactions (AR) in common FD medications

Category	Drug/class	Major reported AE	Incidence of AE/AR
Herbals	STW‐5	Esophagitis, bronchitis, diarrhea, nausea, stomatitis, and abdominal pain^38^	AR: 0.04%^38^ AE: 47.3%^1^
		Liver Failure^81^	<0.0000025%^81^
	RKT	Diarrhea, nausea, headache, γ‐GTP elevation, upper abdominal pain, alanine transaminase elevation, abdominal bloating and discomfort, nasopharyngitis, tinnitus, skin dysesthesia, oral dysesthesia, dizziness, urticarial^1^	AE: 10.8–15.2%^1^ AR: 4.6%^1^
	ALE	Hunger, transient increase in flatulence^64^	45 AEs occurred in 29 patients treated with ALE^64^
	POCO	Nausea, eructation^47^	10.4–19%^1^, ^47^
	ZZKZ	Abdominal pain, diarrhea, nausea^65^	6.63%^65^
Prokinetics	Domperidone	Somnolence^1^	29%^1^
		Reduction of mental acuity^1^	20%^1^
		Sudden cardiac death^88^	4.47 per 1000 pt‐years^88^
	Metoclo‐pramide	Somnolence^1^	49%^1^
		Reduction of mental acuity^1^	33%^1^
		Sudden cardiac death^88^	5.17 per 1000 pt‐years^88^
	Mosapride	Headache, diarrhea, abdominal fullness, palpitation^89^	AE: 21.5%^89^
	Levosulpiride	Amenorrhea, galactorrhea^1^	AE: 18.8%^1^
Acid Suppressants	Proton pump inhibitors	Nasopharyngitis, diarrhea^1^	AR: 5.9–9.4%^1^
		Bone fracture^90^	22%^90^
Muscarinic receptor antagonist	Acotiamide	Headache, diarrhea, nasopharyngitis, increase ALT, γGTP and prolactin levels^1^	AR: 11.5%^1^ AE: 17.9–72.5%^1^

Abbreviations: ALE, Artichoke leaf extract; POCO, peppermint oil and caraway oil; RKT, Rikkunshito; STW‐5, Iberogast; ZZKZ, ZhiZhu KuanZhong.

### 4.3 Formats and consistency of ingredients

Herbal medicines may be presented in formats (e.g., powders, granules, raw herbs) that are less frequently employed in contemporary Western‐style medications (e.g., STW‐5 drop formulation) and which could affect precision of dosing. While some (e.g., peppermint oil) may consist of a single ingredient, many are formulations of a number of herbs (e.g., POCO, STW‐5). Furthermore, the same name has been used to label formulations with different combinations of different herbs. Take the example of the Chinese herbal formula known by the name of *Shugan Jianpi Zhixie* intended for IBS‐D. In a systematic review of RCTs, no fewer than six different concoctions containing different ingredients among them had used the *Shugan Jianpi Zhixie* name.^91^ Thus, for non‐proprietary herbal medicines, because of the possibility of heterogeneity in contents, it would be inappropriate to evaluate these studies together in a meta‐analysis.

### 4.4 Dosage standardization and quality control

For clinical trials, it is essential to achieve consistency of ingredients and composition as these are key items for comparability between studies. Precise quantification of each and every ingredient is also essential not only to ensure a minimum effective dose, but also to avoid toxicity from overdosing. In order to ensure that the pharmacological and pharmaceutical properties of herbal medicine formulations available in the routine clinical setting are not different from that tested in clinical trials, they should, at the minimum, be compliant with established quality standards such as Good Agricultural and Collection Practices (GACP), Good Manufacturing Practices (GMP), and Good Laboratory Practice (GLP). We echo WHO that quality assurance should be the shared responsibility of manufacturers and regulatory bodies.^92^ Thus, concentrations of heavy metals such as Lead, Mercury, Cadmium, and Arsenic, as well as other possible contaminants from pesticide residues and microbes for all plant extracts, need to be monitored at all levels of the production process, from the harvested raw material to the finished herbal product, and should remain within limited boundaries of variability (See Table 4). China, for example, implemented a Green Trade Standards for the Import and Export of Medicinal Plants and Preparations in 2001 that enforced limitation on heavy metals and contaminants in medicinal plant raw materials, decoction pieces, extracts, and their preparations.^93^ A WHO global survey revealed that while majority of the countries have a registration system for herbal medication which includes quality controls, others have identified difficulties including lack of research data, appropriate control mechanisms, and lack of training.^94^

**TABLE 4:** Proposed quality framework for herbal medicines in FGID (adapted from Holtmann et al^83^)

Domain	Measure
Safety	● Appropriate toxicologic assessment of the plant extracts is done; doses tested should provide an enough safety margin (e.g., doses >100‐fold higher compared to clinically used doses). ● Quality assurance of raw materials: (a) Concentrations of active ingredients and/or lead substances are monitored in raw materials to enable adjustments of production process to ensure consistent product qualities and concentration of active ingredients in the marked product (b) Monitoring of potential contaminations (e.g., heavy metals, mold) ● Appropriate pharmacovigilance of products used in the routine clinical setting consistent with the procedures in place for chemically defined treatment
Efficacy	● In vivo and in vitro studies to explore mechanisms of action of the plant extracts in isolation and in combination ● State‐of‐the‐art clinical trials to proof efficacy regarding relevant outcome parameters (symptom reduction, improvement of QoL) paired with studies on mechanisms
Registration, market access	● Formal registration that mirrors chemically defined products for the respective jurisdictions

### 4.5 Translating traditional to contemporary health systems

Many herbal medicines were developed and applied within traditional medicine systems. These may employ a different concept of anatomy and physiology. As an example, in TCM, the diagnostic labels “Spleen Deficiency and Qi Stagnation Syndrome” and “Liver‐Stomach Disharmony Syndrome” are used to describe symptom clusters comprising epigastric fullness, bloating and abdominal pain, and epigastric fullness and pain, respectively.^95^, ^96^ Furthermore, the traditional herbalist employs a more personalized approach whereby the proportion of individual herbs within a compound herbal decoction may be varied according to the individual patient's constitution.

One suggestion that we have is the use of N‐of‐1 trial as a means to marry the robustness of contemporary clinical trial methodology with the personalized medicine approach of traditional herbal medicine. N‐of‐1 trial is a subgroup of the RCT methodology, with a single patient being exposed to double‐blinded, randomized crossover conditions.^97^ The University of Oxford Centre for Evidence‐Based Medicine (CEBM) recently graded N‐of‐1 trials as Level 1 evidence, in the same category as systematic reviews of randomized trials.^98^

## 5 SUMMARY AND CONCLUSIONS

An evaluation of the current treatment landscape in FD reveals important limitations in efficacy, reliable predictors of response, addressing multiple overlapping pathophysiologies. Herbal medicines, either as a singular herb or as a combination of multiple herbs, have frequently been reported to possess properties of affecting simultaneously multiple pathophysiologies. Several herbal formulations that have undergone substantial preclinical and clinical testing with promising results for the treatment of FD are reviewed in more detail. In the background, we also see a growing interest among the general public in using herbal medicines. However, toxicity, efficacy, and standardization of herbal products remain concerns. Therefore, the same scientific rigor that is applied to chemical defined therapies should be applied to the evaluation of herbal therapies at all stages of the development process. We commend herbal medicines as a potential option for the treatment of FD.

## DISCLOSURE

Kok‐Ann Gwee has given scientific advice to Adare and Biocodex, and has been on the speaker bureau of Biocodex, Eisai, and Takeda. Gerald Holtmann has given scientific advice to Allergan, Danone, Bayer, Takeda, and Zeria, and has been on the speaker bureau of Bayer and Gaetz. His organization has received research support from Abbott, Bayer, Boehringer Ingelheim, and Novartis. Jan Tack has given Scientific advice to Adare, AlfaWassermann, Allergan, Christian Hansen, Danone, Grünenthal, Ironwood, Janssen, Kyowa Kirin, Menarini, Mylan, Neutec, Novartis, Noventure, Nutricia, Shionogi, Shire, Takeda, Theravance, Tramedico, Truvion, Tsumura, Zealand, and Zeria pharmaceuticals; has received research support from Shire, Sofar, and Tsumura; and has served on the Speaker bureau for Abbott, Allergan, AstraZeneca, Janssen, Kyowa Kirin, Menarini, Mylan, Novartis, Shire, Takeda, Truvion, and Zeria. Hidekazu Suzuki, has received research support from Daiichi‐Sankyo, EA pharma, Mylan, MSD, Takeda, and Tanabe and has received lecture fee from AstraZeneca, Astellas, Daiichi‐Sankyo, EA pharma, Mylan, Otsuka, and Takeda. Jinsong Liu, Yinglian Xiao, Min‐hu Chen, Xiaohua Hou, Deng‐Chyang Wu, Clarissa Toh, Fang Lu, and Xu‐Dong Tang have no competing interests.

## AUTHOR CONTRIBUTIONS

Guarantor of the article: Kok‐Ann Gwee. Author contributions: KAG and XDT conceptualized the study. KAG and CT performed the research and drafted the manuscript. KAG, GH, HS, JL, YX, DCW, CT, and XDT wrote various sections of the paper. MHC, JT, XH, and FL provided overall advice and direction. Each of the authors participated in working team meetings, contributed to the conceptualization of the report, and took responsibility for specific sections of the report, including literature searches and writing of the section. All authors reviewed and approved the final manuscript.

## Footnotes

^a^ Dietary supplements other than vitamins and minerals.

^b^ TSS: Total Symptom Score, SDS: single dyspepsia symptom; PDSS: postprandial distress syndrome score and CGI: Clinical Global Impression.
